# Automated Spleen Injury Detection Using 3D Active Contours and Machine Learning

**DOI:** 10.3390/e23040382

**Published:** 2021-03-24

**Authors:** Julie Wang, Alexander Wood, Chao Gao, Kayvan Najarian, Jonathan Gryak

**Affiliations:** 1 Department of Electrical Engineering and Computer Science, University of Michigan, Ann Arbor, MI 48109, USA; wangjuli@umich.edu (J.W.); kayvan@med.umich.edu (K.N.); 2 Department of Computational Medicine and Bioinformatics, University of Michigan, Ann Arbor, MI 48109, USA; alexwood2@gmail.com (A.W.); gchao@umich.edu (C.G.); 3 Department of Emergency Medicine, University of Michigan, Ann Arbor, MI 48109, USA; 4 Michigan Center for Integrative Research in Critical Care, University of Michigan, Ann Arbor, MI 48109, USA; 5 Michigan Institute for Data Science, University of Michigan, Ann Arbor, MI 48109, USA

**Keywords:** image segmentation, computer-assisted diagnosis, machine learning, spleen injury detection

## Abstract

The spleen is one of the most frequently injured organs in blunt abdominal trauma. Computed tomography (CT) is the imaging modality of choice to assess patients with blunt spleen trauma, which may include lacerations, subcapsular or parenchymal hematomas, active hemorrhage, and vascular injuries. While computer-assisted diagnosis systems exist for other conditions assessed using CT scans, the current method to detect spleen injuries involves the manual review of scans by radiologists, which is a time-consuming and repetitive process. In this study, we propose an automated spleen injury detection method using machine learning. CT scans from patients experiencing traumatic injuries were collected from Michigan Medicine and the Crash Injury Research Engineering Network (CIREN) dataset. Ninety-nine scans of healthy and lacerated spleens were split into disjoint training and test sets, with random forest (RF), naive Bayes, SVM, *k*-nearest neighbors (*k*-NN) ensemble, and subspace discriminant ensemble models trained via 5-fold cross validation. Of these models, random forest performed the best, achieving an Area Under the receiver operating characteristic Curve (AUC) of 0.91 and an F1 score of 0.80 on the test set. These results suggest that an automated, quantitative assessment of traumatic spleen injury has the potential to enable faster triage and improve patient outcomes.

## 1. Introduction

Blunt spleen injuries account for up to half of all abdominal solid organ injuries. Common causes include road traffic accidents, falls, physical assaults, and sports-related injuries. Multiphasic contrast-enhanced computed tomography (CT) is the standard non-invasive diagnostic tool for injury evaluation of blunt spleen injuries [1], which include lacerations, subcapsular or parenchymal hematomas, active hemorrhage, and vascular injuries. The type and severity of spleen injuries are commonly described based on the Abbreviated Injury Scale (AIS) or the American Association for Trauma (AAST) Organ Injury Scale (OIS). Currently, detection and classification of spleen injuries rely on the manual review of radiologists. This manual process is not only inefficient but also subject to variability based on the reviewer [1,2].

Many computer-assisted diagnosis (CAD) systems have been developed to detect, locate, and assess potential anomalies or injuries to aid radiologists in the diagnostic process. Detection of pathology in the chest, breast, and colon has been the main focus of previous CAD studies [3]. Other extant CAD systems include those that target the brain, liver, skeletal, and vascular systems [3,4,5]. Although there have not been previous studies on CAD systems for the spleen, an automated method for localizing and segmenting the spleen [6] was previously developed by the co-authors of this study. This method can be utilized to segment the region of interest in a CT volume, a prerequisite step to performing spleen injury detection.

Machine learning techniques, including Support Vector Machines (SVM), random forest (RF), logistic regression (LR), and deep learning methods, have been widely applied for analysis of medical images [5]. A critical step in application of machine learning to medical image analysis is the extraction and representation of features salient to the classification or detection task at hand. Different types of features and feature extraction methods have been employed based on the anomaly of interest. Common features used include histogram-based [7,8], shape-based [7,9,10,11], texture-based [12,13,14], region-based [10,15], and bag-of-words features [15], among others.

**Figure 1:**
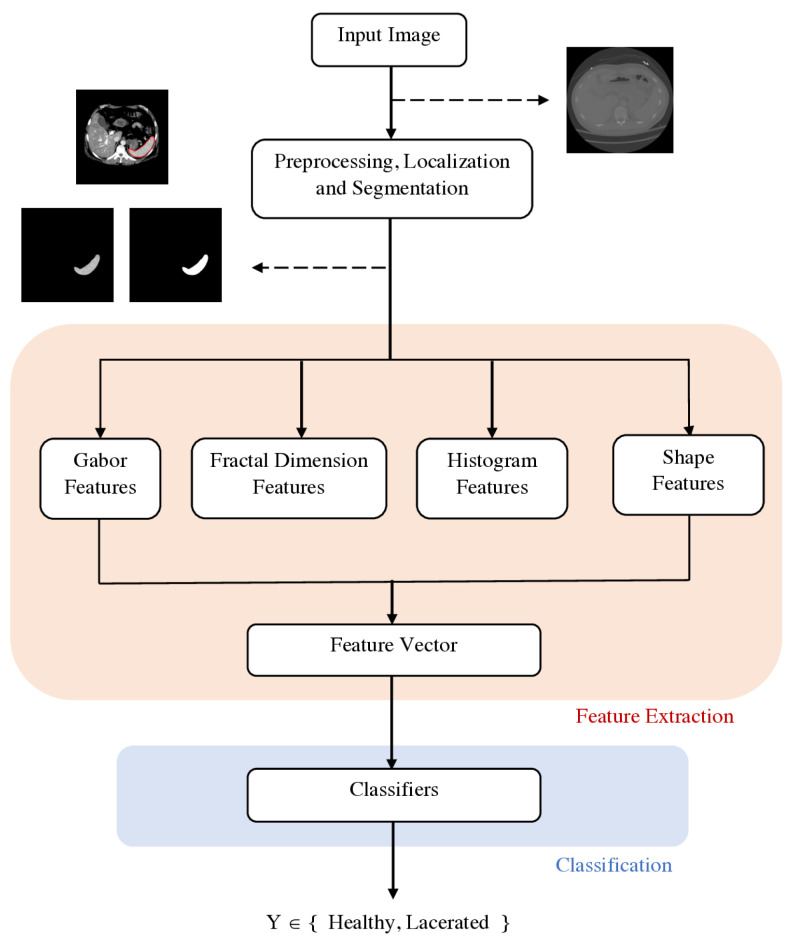
A schematic diagram of the proposed method.

In this paper, we propose a supervised classification scheme to discriminate lacerated spleens from healthy controls, a schematic diagram that is presented in Figure 1. Lacerations were chosen for study as they are major types of blunt spleen injury that can be readily observed from contrast-enhanced CT, appearing as linear or branching regions extending from the capsular surface of the spleen and often disrupting the smooth splenic contour [1]. CT scans from patients experiencing traumatic injuries were collected from the Michigan Medicine and the Crash Injury Research Engineering Network (CIREN) dataset [16]. Healthy and lacerated spleens within CT scans from 99 patients were automatically segmented using a previously developed method [6]. From the segmented spleen region, various features were extracted: statistical histogram-based features including Rényi entropy; shape-based feature including fractal dimension [17], whose generalized version is directly related to Rényi entropy [18]; and texture-based features. The performance of five machine learning models: RF, naive Bayes, SVM, *k*-nearest neighbors (*k*-NN) ensemble, and subspace discriminant ensemble, were trained using 5-fold cross-validation. On a distinct test set, RF was the best performing classifier, achieving an Area Under the receiver operating characteristic Curve (AUC) of 0.91 and F1 score of 0.80. This study demonstrates the potential for such an automated injury assessment method to reduce physician workload and improve patient outcomes by enabling faster injury triage.

## 2. Materials and Methods

### 2.1. Dataset

CT scans used in this study were obtained from Michigan Medicine patients who experienced traumatic abdominopelvic injuries under an IRB-approved retrospective study. Patient consent was waived by the IRB as the research involved no more than minimal risk to the subjects. Additional training data were obtained from the Crash Injury Research Engineering Network (CIREN) dataset [16] containing CT volumes for patients who experienced traumatic injuries in a motor vehicle accident. Each patient CT scan used in this study contained an axial abdominopelvic volume, comprised of between 42 and 122 slices of 5 mm thickness from the heart to the pelvic region. Samples with artifacts around the spleen region were removed.

A total of 99 CT scans, one per patient, were used in this study, consisting of 54 healthy spleen samples and 45 lacerated spleen samples. The lacerated samples are categorized by the Abbreviated Injury Scale (AIS) and the Organ Injury Scale (OIS). Of the 45 lacerated spleen samples, the distribution of injury is as follows: OIS grade I or II (AIS = 2): 15, OIS grade III (AIS = 3): 16, OIS grade IV (AIS = 4): 10, OIS grade V (AIS = 5): 4.

The previously developed spleen segmentation method utilized in this study [6] also made use of the Michigan Medicine and CIREN datasets. In that study, CT scans from 147 patients (one scan per patient) were used to train and test automated spleen segmentation on patients with healthy spleens. The training set was composed of 108 patients, 65 from Michigan Medicine, and 43 from CIREN, with a disjoint test set containing 39 CT scans, 21 from Michigan Medicine, and 18 from CIREN. The patients utilized for training in the prior segmentation study are distinct from those used in this study for training spleen injury detection.

### 2.2. Spleen Segmentation

Segmentations of the spleen were obtained from each abdominopelvic CT volume using a previously developed fully automated spleen localization and segmentation method [6]. Preprocessing was first applied to the images in order to remove noise through standard and local contrast adjustment, as well as the application of image denoising filters. Localization then utilized machine learning methods to identify a small region within the spleen as a seed mask. Segmentation was then performed via a series of reinitialized active contours using the established seed mask.

**Figure 2:**
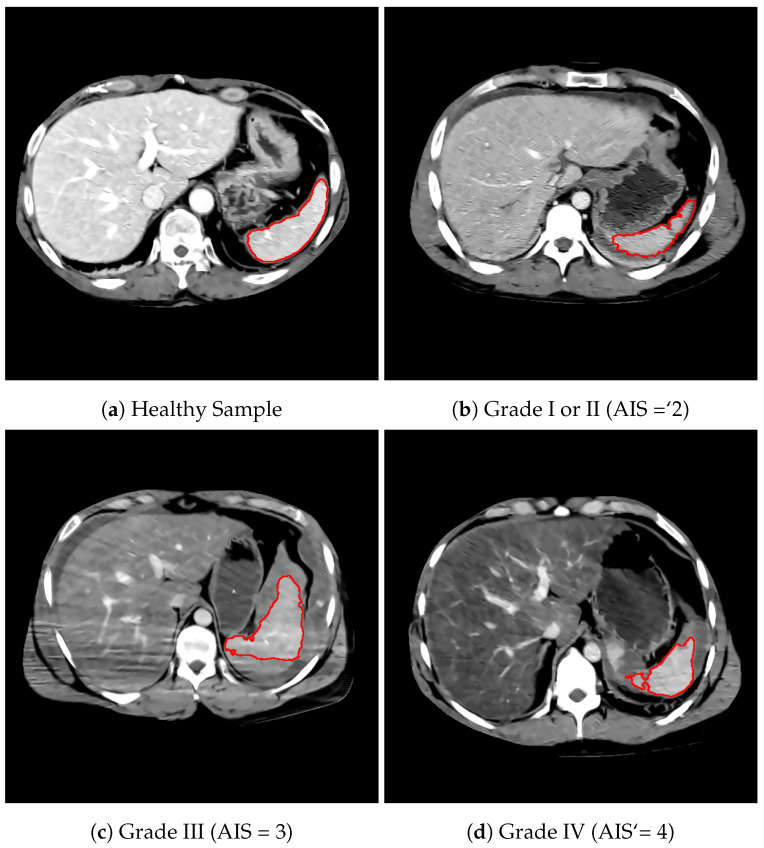
Segmentation of healthy and lacerated spleens.

Segmentations that resulted in a total segmented spleen volume of less than 80 cm${}^{2}$ were considered segmentation errors. This occurred in 6 out of 99 cases, and these samples were removed from the dataset. Manual annotations reviewed by an expert radiologist were obtained for 36 healthy samples as well as one lacerated sample. The segmentation method achieved an average Dice score of 0.87, excluding segmentation errors. Sample segmentations of healthy and lacerated spleens are illustrated in Figure 2.

### 2.3. Feature Extraction

In this study, four types of features—histogram features, fractal dimension features, Gabor features, and shape features—were extracted to train classifiers capable of discriminating injured spleens from healthy controls. Histogram and Gabor features were used to represent and discriminate textures within the spleen segmentation, while fractal and shape analyses were applied to characterize the spleen contour.

#### 2.3.1. Histogram Features

The histogram of an image is a plot of the intensity values of a color channel against the number of pixels at that value. The shape of the histogram provides information regarding the contrast and brightness of the image [19]. Five statistical and information-theoretic features of the histogram were extracted from the data for this analysis: mean, variance, skewness, kurtosis, and Rényi entropy. Mean denotes the average intensity level, while variance represents the variation of intensities around the mean. Skewness measures the asymmetry of the data about the mean and kurtosis specifies whether the distributions are flat or peaked relative to a normal distribution. Additionally, entropy measures the disorder in the image based on the distribution of intensity levels.

#### 2.3.2. Fractal Dimension Analysis

Fractals are mathematical sets with high degrees of geometrical complexity capable of modeling irregular, complex shapes [20]. Fractal features have been widely applied in texture and shape analyses of images, including medical images [9,21] to characterize the irregularity of physical structures.

Fractal dimension ($D_{f}$) is one of the most important fractal features and provides a quantitative measure of the coarseness of an image. Since lacerated spleens generally display an irregular [2], jagged contour as compared to healthy spleens (see Figure 2), the fractal dimension of binary segmentation images was calculated as a shape-based feature. Both the fractal dimension of the segmentation perimeter as well as the segmentation area were extracted.

In this study, the widely used box counting method [17] was employed to estimate $D_{f}$ for each binary image of segmentation, after which the fractal dimension $D_{f}$ was calculated for each frame in the CT volume containing the segmented spleen. Let N(r) denote the number of boxes with fixed side length *r* necessary to cover the positive pixels of the segmentation. The box-counting method iteratively calculates N(r) for each *r* of 1,2,3,...,512 pixels. $D_{f}$ is then calculated by fitting logN(r) to a linear function of logr by the least squares error method.

#### 2.3.3. Gabor Features

A Gabor filter is a linear filter often used for edge detection. Gabor filter-based features are commonly used to represent and discriminate textures in images and are captured from responses of images convolved with Gabor filters. A two-dimensional Gabor filter is a Gaussian kernel function modulated by a complex sinusoidal plane wave, and can be defined as follows:

(1) $$\begin{array}{cc} g(x,y;\lambda ,\theta ,\psi ,\sigma ,\gamma ) & =\exp\left(-\frac{x^{\prime 2}+\gamma^{2}y^{\prime 2}}{2\sigma^{2}}\right)\exp\left(i(2\pi \frac{x^{\prime }}{\lambda }+\psi )\right)\\ x^{\prime } & =x\cos\theta +y\sin\theta \\ y^{\prime } & =-x\sin\theta +y\cos\theta \end{array}$$

In Equation (1), λ is the wavelength of the sinusoidal factor, θ is the orientation of the normal to the parallel stripes of a Gabor function, ψ is the phase offset, σ is the standard deviation of the Gaussian envelope, and γ is the spatial aspect ratio [22,23].

In this study, a filter bank of 40 Gabor filters in 5 scales and 8 orientations was employed. From the response matrices, two types of Gabor features were extracted: local energy and mean amplitude. Local energy is calculated by the sum of the squared values in each response matrix. Mean amplitude captures the response amplitude for each response matrix by taking the sum of absolute values in each matrix.

#### 2.3.4. Shape Features

Values of circularity, eccentricity, orientation, and the difference between the segmented area and its convex area were extracted to characterize the shape of the segmented spleen. Circularity, calculated as

(2) $$\left(4*Area*\pi \right)/\left(Perimeter^{2}\right),$$

captures the roundness of objects; a perfect circle would have a circularity of 1. Eccentricity is the ratio of the distance between the foci of an ellipse and its major axis length. Orientation was calculated as the angle between the *x*-axis and the major axis of an ellipse. In addition, finally, the convex area is the area of the convex hull of the region, defined as the smallest convex set that contains the original region. The difference between this area and the original segmented area was also extracted.

### 2.4. Classification

#### 2.4.1. Training

Ninety-three classification samples were randomly separated into training and test sets, respectively, comprising 80% and 20% of the samples, with the relative number of injury and healthy samples being balanced. As only one CT scan per patient was utilized in this study, patients and their respective scans were exclusively assigned to either the training or test set.

5-fold cross validation was employed during the training phase to select models with low variance and low bias. The training set was divided into 5 folds of roughly equal size. The classifier was then trained on 4 folds and tested on the remaining fold. Validation accuracy, AUC, and the associated standard deviations were used to select models.

#### 2.4.2. Model Selection

Five models—RF, naive Bayes, SVM, *k*-NN ensemble, and subspace discriminant ensemble—were selected based on validation performance during the training phase as reported in Table 1. Of the five models, RF performed the best on the training set with an AUC of 0.91.

RF, naive Bayes, and SVM are all popular supervised learning models used for analysis on medical images [5]. Naive Bayes is a probabilistic classifier applying Bayes theorem with an assumption of feature pairwise independence given class values. Ensemble learning combines several classifiers to improve prediction performance. RF is an ensemble learner that leverages multiple decision trees to produce a more accurate and stable prediction. Subspace discriminant ensemble [24] employs the linear discriminant analysis (LDA) scheme for a specific discriminant subspace of low dimension. The *k*-NN ensemble employed in this study uses the Random Space method with *k*-NN learners.

Deep learning, and more specifically the application of convoluted neural networks (CNN) to image analysis, has achieved great success in recent years [25]. To assess the validity of the hand-crafted features proposed in this study, an end-to-end deep learning method was evaluated along with the traditional machine learning models. A pre-trained CNN, ResNet-50 [26], was used for feature extraction on the segmented CT volumes, with subsequent classification performed by a Long Short-term Memory (LSTM) artificial recurrent neural network (RNN). This combination of CNN for slice-wise feature extraction and LSTM for spatial information extraction across the CT volume has been successful in previous injury detection studies, including classification of intracranial hemorrhage [27,28], lung cancer [29], as well as liver and brain tumors [30]. The goal of this approach is to leverage 2D models pre-trained on the ImageNet dataset [31] while still accounting for spatial information between slices in the 3D volume. ResNet-50 was selected for feature extraction because of its relatively higher accuracy and lower number of parameters (23 M) compared to other architectures commonly used for medical image analysis, such as AlexNet (62 M parameters) and VGGNet (138 M parameters).

Feature extraction was performed by ResNet-50 on each slice of the segmented CT, which were cropped to reduce blank space surrounding the region of interest. An LSTM model was then employed to perform classification on the extracted features across each patient’s CT sequence.

## 3. Results

### 3.1. Classifier Performance

The trained classifiers were evaluated on the test set, with the resulting accuracy, sensitivity, specificity, F1, and AUC reported in Table 2. The RF model achieved the best classification performance with an AUC of 0.91 and an F1 of 0.80. Overall, testing loss and accuracy are consistent with 5-fold cross validation results on the training set, demonstrating the generalizability of the proposed method on unseen data. No over-fitting seemed to occur on any of the models reported.

### 3.2. Comparison against Deep Learning

A comparison between the RF classifier performance and the deep learning method is shown in Table 3. The RF classifier trained with hand-crafted features demonstrated better performance than the deep learning method, with RF achieving an AUC of 0.91 while the deep learning method achieved an AUC of 0.72. The lower deep learning performance is likely due to the small sample size available in this study, as deep learning methods require large datasets to minimize over-fitting and achieve good performance [25,32]. These results demonstrate that hand-crafted features using domain knowledge can overcome sample size limitations.

### 3.3. Leave-One-Site-Out Analysis

This study utilizes two different datasets—the internal Michigan Medicine dataset and the public CIREN dataset. A leave-one-site-out analysis was performed to evaluate the cross-site generalizability of the proposed method.

To achieve an 80% to 20% training/test split, the Michigan Medicine dataset, containing a total of 54 samples, was used as the training set while 14 CIREN samples were used as the test set. The 14 CIREN test samples were randomly stratified based on injury grade. The best performing classifier from Section 2.4.2, RF, was trained on the Michigan Medicine samples and tested on the CIREN samples. Performance metrics of the classifier are reported in Table 4.

The RF classifier achieved good performance on the cross-site generalizability assessment, with an AUC of 0.91 and a F1 of 0.71. Compared to the performance on the mixed-site test set, the classifier achieved the same AUC but lower F1, accuracy, and sensitivity. This performance difference is likely affected by the limited sample size used to train the classifier as only one dataset is utilized. Overall, the performance of the classifier demonstrates that the proposed method is relatively robust against variability stemming from differences in the data from two different sites.

## 4. Discussion

RF outperformed other classifiers on both the training and test set, which is consistent with its popularity among many previous medical image analysis studies [33,34,35,36]. Several features of RF may contribute to its higher performance on medical images—RFs are suited for high predictor dimension relative to sample size, they inherently perform feature selection, and they generalize well to regions of the feature space with sparse data [34,35].

Table 5 reports the classification accuracy of RF by injury grade on both the training and test sets. Although the RF classifier correctly classified the majority of samples across all injury grades, most incorrect classifications occurred within mildly or moderately injured samples (AIS = 2, 3). High classification accuracy is seen among healthy samples and more severe samples (AIS = 4, 5). Lower accuracy and higher variance were achieved for all injury grades as compared to healthy samples, likely due to the smaller number of samples within each individual injury grade compared to the healthy dataset. Despite the lower performance on less severe cases, the proposed method performs well on severe cases, demonstrating the potential to increase injury triage efficiency in real-world applications.

**Figure 3:**
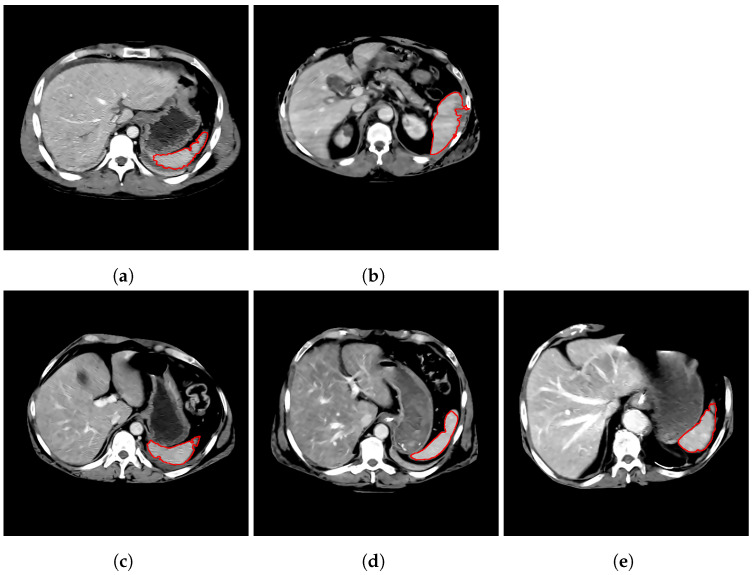
Classification results. (**a**,**b**) lacerated (AIS = 2) samples correctly classified as lacerated; (**c**) lacerated (AIS = 2) sample incorrectly classified as healthy; (**d**,**e**) healthy samples incorrectly classified as lacerated.

Common misclassifications included classification of a mildly or moderately injured sample as healthy and classification of a healthy sample as lacerated, as illustrated in Figure 3. A lacerated sample with lower injury severity misclassified as healthy is likely caused by a relatively smooth segmentation contour (Figure 3c), which may be the result of imperfect segmentation of the lacerated region and/or a lower degree of laceration. Healthy samples misclassified as lacerated were often due to noise in the original image, which produces misleading segmentations or irregular contour shapes (Figure 3d,e).

Existence of a small portion of samples with localization errors likely led to lower model performance due to imperfect or erroneous segmentations. Image resolution and noise are likely contributing factors to imperfect localization and segmentation results. Previous studies have shown that image thickness is inversely related to image noise but directly related to image resolution [37]. In order, 5 mm CT slices were utilized in this study, which has worked relatively well due to its lower image noise compared to thinner slices. However, 5 mm slices have a lower resolution, decreasing diagnostic content and the proposed method’s ability to detect small lesions. Although not available in the datasets utilized in this study, 3 mm slices may strike an ideal balance between minimizing image noise and maximizing image resolution and can be explored in the future [37].

Future work will focus on refinement of the segmentation method to improve classification accuracy in lower severity cases. Additional pre- and post-processing of in the segmentation method can be introduced to reduce noise and increase discrimination between healthy and mildly lacerated spleen. Incorporation of more samples in each injury grade may increase classifier performance and support extension of the current binary classification to multi-class classification on different injury grades, providing additional clinical use cases. Finally, although this study focuses on spleen lacerations, future work should generalize to other blunt spleen injuries, including hematomas and hemorrhages.

## 5. Conclusions

In this study, an automated method for detecting spleen lacerations in CT scans was proposed. The classification scheme was built upon a previously developed localization and segmentation process [6], and used histogram, Gabor filters, fractal dimension, and shape features to distinguish lacerated spleens from healthy controls. Classifiers examined were RF, naive Bayes, SVM, *k*-NN ensemble, subspace discriminant ensemble, and a CNN-based architecture. The RF method outperformed other models in discriminating between lacerated and healthy spleens, achieving an AUC of 0.91 and an F1 of 0.80. Additionally, a leave-one-site-out analysis was performed that demonstrated the method’s robustness against variability stemming from differences in the data from two different sites. Results from this study demonstrate the potential for automated, quantitative assessment of traumatic spleen injury to increase triage efficiency and improve patient outcomes. Future work will focus on improving classifier accuracy in less severe cases, extension of the method to support multi-class classification based on injury grade, and generalization to other types of blunt spleen injuries.

## Figures and Tables

**Table 1:** Mean and standard deviation (SD) of performance metrics for spleen injury classification from 5-fold cross validation on the training set. The highest value for each performance metric is **bolded** while the lowest SD is *italicized*.

Metric	RF	Naive Bayes	SVM	*k*-NN	Subspace Discriminant
Accuracy	**0.83** (*0.10*)	0.71 (0.11)	0.73 (*0.10*)	0.73 (*0.10*)	0.67 (*0.10*)
Sensitivity	**0.77** (*0.16*)	0.66 (0.17)	0.61 (0.17)	0.56 (0.18)	0.44 (0.19)
Specificity	**0.89** (0.12)	0.75 (0.15)	0.84 (0.13)	**0.89** (*0.11*)	0.87 (0.13)
F1	**0.81** (*0.12*)	0.68 (0.13)	0.67 (0.14)	0.65 (0.16)	0.54 (0.18)
AUC	**0.91** (*0.08*)	0.75 (0.12)	0.81 ( 0.10)	0.84 (0.10)	0.77 (0.13)

**Table 2:** Performance metrics for spleen injury classification on the test set. The highest value for each performance metric is **bolded**.

Metric	RF	Naive Bayes	SVM	*k*-NN	Subspace Discriminant
Accuracy	**0.83**	0.70	0.71	0.75	0.64
Sensitivity	**0.76**	0.63	0.56	0.59	0.40
Specificity	**0.89**	0.76	0.85	0.88	0.85
F1	**0.80**	0.66	0.64	0.68	0.50
AUC	**0.91**	0.74	0.80	0.84	0.76

**Table 3:** Performance metrics for the RF classifier trained using hand-crafted features and for the deep learning method. The highest value for each performance metric is **bolded**.

Metric	RF (Hand-Crafted)	ResNet + LSTM (Deep Learning)
Accuracy	**0.83**	0.79
Sensitivity	**0.76**	0.67
Specificity	0.89	**0.90**
F1	**0.80**	0.75
AUC	**0.91**	0.72

**Table 4:** Performance metrics for the RF classifier trained on Michigan Medicine samples and tested on CIREN samples.

Metric	RF
Accuracy	0.75
Sensitivity	0.59
Specificity	0.94
F1	0.71
AUC	0.91

**Table 5:** RF classification accuracy by injury grades. The mean accuracy and standard deviation (SD) across 5-fold cross validation on the training set, as well as the mean accuracy on the test set are reported.

Injury Grade	Training Accuracy	Testing Accuracy
Healthy	0.89 (0.12)	0.89
AIS = 2	0.72 (0.30)	0.70
AIS = 3	0.74 (0.27)	0.78
AIS = 4, 5	0.88 (0.22)	0.79

## Data Availability

Two datasets were employed in this study—the Crash Injury Research Engineering Network (CIREN) dataset and an internal dataset collected from Michigan Medicine. CIREN is a public dataset that is available for download at https://www.nhtsa.gov/research-data/crash-injury-research (accessed on 1 February 2021). Data collected from Michigan Medicine can be made available to external entities under a data use agreement with the University of Michigan.

## References

[1] Shi H., Teoh W., Chin F., Tirukonda P., Cheong S., Yiin R. (2019). CT of blunt splenic injuries: What the trauma team wants to know from the radiologist. Clin. Radiol..

[2] Hassan R., Aziz A.A., Ralib A.R.M., Saat A. (2011). Computed tomography of blunt spleen injury: A pictorial review. Malays. J. Med. Sci. MJMS.

[3] Zhang Z., Sejdić E. (2019). Radiological images and machine learning: Trends, perspectives, and prospects. Comput. Biol. Med..

[4] Doi K. (2007). Computer-aided diagnosis in medical imaging: Historical review, current status and future potential. Comput. Med Imaging Graph..

[5] Syeda-Mahmood T. (2018). Role of big data and machine learning in diagnostic decision support in radiology. J. Am. Coll. Radiol..

[6] Wood A., Soroushmehr S.R., Farzaneh N., Fessell D., Ward K.R., Gryak J., Kahrobaei D., Najarian K. Fully Automated Spleen Localization In addition, Segmentation Using Machine Learning In addition, 3D Active Contours. Proceedings of the 2018 40th Annual International Conference of the IEEE Engineering in Medicine and Biology Society (EMBC).

[7] Shi X., Cheng H.D., Hu L., Ju W., Tian J. (2010). Detection and classification of masses in breast ultrasound images. Digit. Signal Process..

[8] Dhanalakshmi K., Rajamani V. (2013). An intelligent mining system for diagnosing medical images using combined texture-histogram features. Int. J. Imaging Syst. Technol..

[9] Lee W.L., Chen Y.C., Hsieh K.S. (2003). Ultrasonic liver tissues classification by fractal feature vector based on M-band wavelet transform. IEEE Trans. Med. Imaging.

[10] Xu Y., Lin L., Hu H., Yu H., Jin C., Wang J., Han X., Chen Y.W. (2016). Combined density, texture and shape features of multi-phase contrast-enhanced CT images for CBIR of focal liver lesions: A preliminary study. Innovation in Medicine and Healthcare 2015.

[11] Dhara A.K., Mukhopadhyay S., Dutta A., Garg M., Khandelwal N. (2016). A combination of shape and texture features for classification of pulmonary nodules in lung CT images. J. Digit. Imaging.

[12] Zhu X., He X., Wang P., He Q., Gao D., Cheng J., Wu B. (2016). A method of localization and segmentation of intervertebral discs in spine MRI based on Gabor filter bank. Biomed. Eng. Online.

[13] Wu C.C., Lee W.L., Chen Y.C., Lai C.H., Hsieh K.S. (2012). Ultrasonic liver tissue characterization by feature fusion. Expert Syst. Appl..

[14] Lee W.L. (2013). An ensemble-based data fusion approach for characterizing ultrasonic liver tissue. Appl. Soft Comput..

[15] Alkhawlani M., Elmogy M., Elbakry H. (2015). Content-based image retrieval using local features descriptors and bag-of-visual words. Int. J. Adv. Comput. Sci. Appl..

[16] U.S. Department of Transportation, National Highway Traffic Safety Administration (NHTSA) (2017). Crash Injury Research Engineering Network. https://www.nhtsa.gov/research-data/crash-injury-research.

[17] Keller J.M., Crownover R.M., Chen R.Y. (1987). Characteristics of natural scenes related to the fractal dimension. IEEE Trans. Pattern Anal. Mach. Intell..

[18] Zmeskal O., Dzik P., Vesely M. (2013). Entropy of fractal systems. Comput. Math. Appl..

[19] Sergyan S. Color histogram features based image classification in content-based image retrieval systems. Proceedings of the 2008 6th International Symposium on Applied Machine Intelligence and Informatics.

[20] Mandelbrot B.B. (1983). The Fractal Geometry of Nature.

[21] Chen C.C., DaPonte J.S., Fox M.D. (1989). Fractal feature analysis and classification in medical imaging. IEEE Trans. Med. Imaging.

[22] Zheng D., Zhao Y., Wang J. Features extraction using a Gabor filter family. Proceedings of the Sixth IASTED International Conference, Signal and Image Processing.

[23] Haghighat M., Zonouz S., Abdel-Mottaleb M. (2015). CloudID: Trustworthy cloud-based and cross-enterprise biometric identification. Expert Syst. Appl..

[24] Ashour A.S., Guo Y., Hawas A.R., Xu G. (2018). Ensemble of subspace discriminant classifiers for schistosomal liver fibrosis staging in mice microscopic images. Health Inf. Sci. Syst..

[25] Lee J.G., Jun S., Cho Y.W., Lee H., Kim G.B., Seo J.B., Kim N. (2017). Deep learning in medical imaging: General overview. Korean J. Radiol..

[26] He K., Zhang X., Ren S., Sun J. Deep Residual Learning for Image Recognition. Proceedings of the 2016 IEEE Conference on Computer Vision and Pattern Recognition (CVPR).

[27] Burduja M., Ionescu R.T., Verga N. (2020). Accurate and Efficient Intracranial Hemorrhage Detection and Subtype Classification in 3D CT Scans with Convolutional and Long Short-Term Memory Neural Networks. Sensors.

[28] Nguyen N.T., Tran D.Q., Nguyen N.T., Nguyen H.Q. (2020). A CNN-LSTM Architecture for Detection of Intracranial Hemorrhage on CT scans. arXiv.

[29] Marentakis P., Karaiskos P., Kouloulias V., Kelekis N., Argentos S., Oikonomopoulos N., Loukas C. (2021). Lung cancer histology classification from CT images based on radiomics and deep learning models. Med. Biol. Eng. Comput..

[30] Kutlu H., Avcı E. (2019). A novel method for classifying liver and brain tumors using convolutional neural networks, discrete wavelet transform and long short-term memory networks. Sensors.

[31] Russakovsky O., Deng J., Su H., Krause J., Satheesh S., Ma S., Huang Z., Karpathy A., Khosla A., Bernstein M. (2015). Imagenet large scale visual recognition challenge. Int. J. Comput. Vis..

[32] Luo C., Li X., Wang L., He J., Li D., Zhou J. How Does the Data set Affect CNN-based Image Classification Performance?. Proceedings of the 2018 5th International Conference on Systems and Informatics (ICSAI).

[33] Tang T.T., Zawaski J.A., Francis K.N., Qutub A.A., Gaber M.W. (2019). Image-based classification of tumor type and growth rate using machine learning: A preclinical study. Sci. Rep..

[34] Nedjar I., EL HABIB DAHO M., Settouti N., Mahmoudi S., Chikh M.A. (2015). Random forest based classification of medical x-ray images using a genetic algorithm for feature selection. J. Mech. Med. Biol..

[35] Geremia E., Clatz O., Menze B.H., Konukoglu E., Criminisi A., Ayache N. (2011). Spatial decision forests for MS lesion segmentation in multi-channel magnetic resonance images. NeuroImage.

[36] Lebedev A., Westman E., Van Westen G., Kramberger M., Lundervold A., Aarsland D., Soininen H., Kłoszewska I., Mecocci P., Tsolaki M. (2014). Random Forest ensembles for detection and prediction of Alzheimer’s disease with a good between-cohort robustness. Neuroimage Clin..

[37] Alshipli M., Kabir N.A. (2017). Effect of slice thickness on image noise and diagnostic content of single-source-dual energy computed tomography. J. Phys. Conf. Ser. IOP Publ..

